# Blunt cardiac injury due to trauma associated with snowboarding: a case report

**DOI:** 10.1186/s13256-017-1242-2

**Published:** 2017-03-25

**Authors:** Fuminori Yamaji, Hideshi Okada, Yasuhiro Nakajima, Kodai Suzuki, Takahiro Yoshida, Yosuke Mizuno, Haruka Okamoto, Yuichiro Kitagawa, Taku Tanaka, Shiho Nakano, Sho Nachi, Tomoaki Doi, Keisuke Kumada, Shozo Yoshida, Narihiro Ishida, Katsuya Shimabukuro, Hiroaki Ushikoshi, Izumi Toyoda, Kiyoshi Doi, Shinji Ogura

**Affiliations:** 1 Department of Emergency and Disaster Medicine, Gifu University Graduate School of Medicine, 1-1 Yanagido, Gifu, 501-1194 Japan; 2 General and Cardiothoracic Surgery, Gifu University Graduate School of Medicine, 1-1 Yanagido, Gifu, 501-1194 Japan

**Keywords:** Blunt cardiac injury, Snowboarding trauma

## Abstract

**Background:**

Cardiac trauma is associated with a much higher mortality rate than injuries to other organ systems, even though cardiac trauma is identified in less than 10% of all trauma admissions. Here we report blunt trauma of the left atrium due to snowboarding trauma.

**Case presentation:**

A 45-year-old Asian man collided with a tree while he was snowboarding and drinking. He lost consciousness temporarily. An air ambulance was requested and he was transported to an advanced critical care center. On arrival, a pericardial effusion was detected by a focused assessment with sonography for trauma. His presenting electrocardiogram revealed normal sinus rhythm and complete right bundle branch block. Laboratory findings included a white blood cell count of 13.5 × 10^3^/μl, serum creatine kinase level of 459 IU/l, and creatine kinase–myocardial band level of 185 IU/l.

Enhanced computed tomography showed a large pericardial effusion and bleeding from his left adrenal gland. There were no pelvic fractures. A diagnosis of cardiac tamponade due to blunt cardiac injury and left adrenal injury due to blunt trauma was made. Subsequently, emergency thoracic surgery and transcatheter arterial embolization of his left adrenal artery were performed simultaneously. A laceration of the left atrial appendage in the lateral wall of his left ventricle was detected intraoperatively and repaired. His postoperative course progressed favorably, although a pericardial effusion was still detected on chest computed tomography on hospital day 35. His electrocardiogram showed normal sinus rhythm and the complete right bundle branch block pattern changed to a narrow QRS wave pattern. He was discharged on hospital day 40.

**Conclusions:**

The present case report illustrates two points: (1) severe injuries resulted from snowboarding, and (2) complete right bundle branch block was caused by blunt cardiac injury. The present report showed blunt trauma of the left atrium with complete right bundle branch block as an electrocardiogram change due to snowboarding trauma. To detect cardiac trauma in snowboarding accidents, an examination of an electrocardiogram is required in all patients who might have a bruised chest.

## Background

Cardiac trauma is associated with a much higher mortality rate than injuries to other organ systems, even though cardiac trauma is identified in less than 10% of all trauma admissions [1]. Owing to its anterior location, the right ventricle is the most commonly injured chamber; it is injured in 40% of trauma victims. On the other hand, left atrial injuries are the least common because of their location.

Snowboarding is remarkable for its dramatic rise in popularity and there is an increasing incidence in trauma ranging from minor injury to severe head injury, as well as multiple injuries [2]. The most common injuries were to the wrist, the hand, and the head [3]. However, there are few reports that cardiac trauma is caused in snowboarding trauma [4]. Here we report blunt trauma of the left atrium due to trauma associated with snowboarding.

## Case presentation

A 45-year-old Asian man collided with a tree while snowboarding and drinking. He temporarily lost consciousness; therefore, an air ambulance was requested. His level of consciousness improved, and his Glasgow Coma Scale (GCS) score was 15 when the air ambulance physicians reached him. However, his condition subsequently deteriorated. His radial artery pulse was not palpable bilaterally and his carotid artery pulse was faint. Subcutaneous emphysema and flail chest were not detected and there was no active bleeding from the site of injury. A physical examination revealed tachypnea with a respiratory rate (RR) of 30 breaths per minutes. Oxygen saturation (SpO_2_) based on pulse oximetry was not measurable. Jugular venous distention was not observed and there was no pelvic instability. A focused assessment with sonography for trauma (FAST) was performed. Intraperitoneal hemorrhage was not detected and there was no pericardial effusion.

During air ambulance transportation, his blood pressure became unstable. On arrival at our advanced critical care center, his heart rate was 95 beats per minute and blood pressure was 75/50 mmHg. A pericardial effusion was detected when FAST was performed again.

**Fig. 1:**
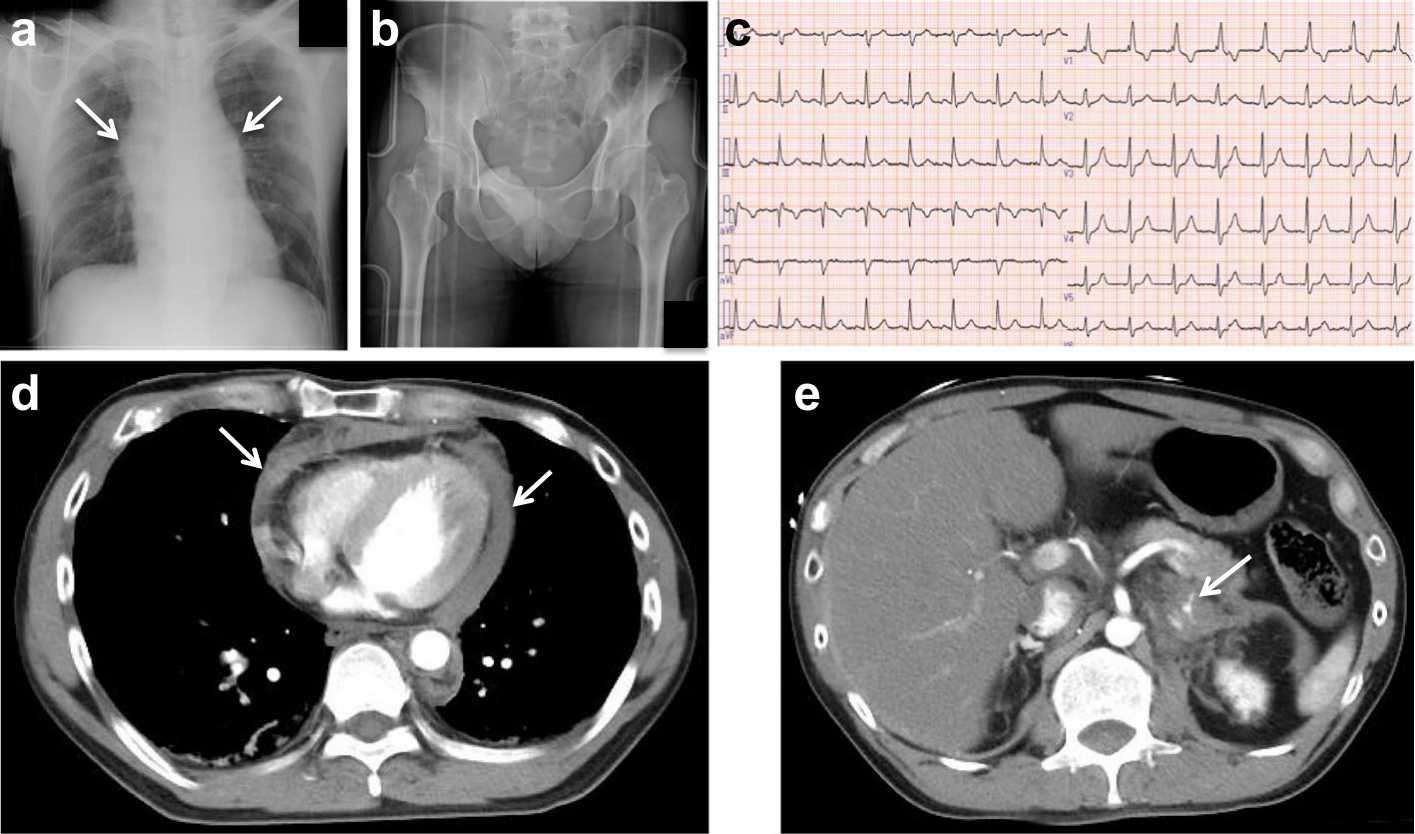
**a** An anteroposterior chest radiograph showed an enlarged mediastinum (*arrows*). **b** A pelvis radiograph showed no obvious pelvic fractures. **c** The electrocardiogram on arrival at our hospital showed complete right bundle block and normal sinus rhythm. **d** Two-dimensional enhanced axial chest computed tomography showed a large pericardial effusion (*arrows*). Since the pericardium had a radiodensity of 30 to 50 Hounsfield units, it was thought that the pericardial effusion was bloody. **e** Two-dimensional enhanced axial abdominal computed tomography showed bleeding from left adrenal gland (*arrow*)

Anteroposterior chest radiographs showed an enlarged mediastinum (Fig. 1a). No fractures were detected on radiographs of his pelvis (Fig. 1b). His presenting electrocardiogram (ECG) revealed normal sinus rhythm and complete right bundle branch block (CRBBB; Fig. 1c).

Arterial blood gas analysis on 100% oxygen revealed a pH of 6.958, partial pressure of oxygen in arterial blood (PaO_2_) of 342 mmHg, partial pressure of carbon dioxide in arterial blood (PaCO_2_) of 31.3 mmHg, bicarbonate (HCO_3_^−^) of 6.7 mmol/l, base excess of −25.3 mmol/l, and lactate of 157 mg/dl. Laboratory findings included a white blood cell count of 13.5 × 10^3^/μl, serum creatine kinase (CK) level of 459 IU/l, and CK–myocardial band (CK-MB) level of 185 IU/l.

**Fig. 2:**
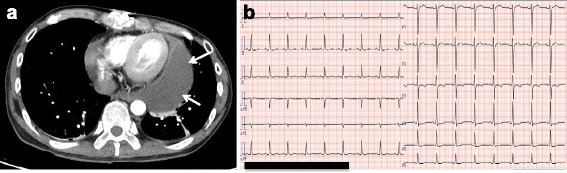
**a** Two-dimensional axial enhanced chest computed tomography on hospital day 35. An effusion remained in the pericardium (*arrows*). **b** Electrocardiography on hospital day 35 revealed a narrow QRS pattern with normal sinus rhythm

For a systemic evaluation, enhanced whole body computed tomography (CT) was then performed. It revealed: a large pericardial effusion (Fig. 1d); bleeding from his left adrenal gland and abdominal aorta (Fig. 1e); fractures of his left fourth, sixth, and seventh ribs; and no pelvic fractures. Therefore, we diagnosed cardiac tamponade due to blunt cardiac injury, as well as left adrenal injury, and abdominal aortic dissection due to blunt trauma. Subsequently, emergency thoracic surgery and transcatheter arterial embolization of his left adrenal artery were performed simultaneously. A laceration of the left atrial appendage was detected in the lateral wall of his left ventricle during surgery, which was thought to be responsible for the cardiac tamponade. We repaired the laceration. His postoperative course progressed favorably, although pericardial effusion was still detected on chest CT (Fig. 2a) and echocardiography on hospital day 35. His ECG showed normal sinus rhythm, and the CRBBB pattern changed to a narrow QRS wave pattern (Fig. 2b). He was discharged on hospital day 40.

## Discussion

The present case illustrates two important points. The first is that severe injury resulted from a snowboarding accident. Like some other winter sports, snowboarding comes with a level of risk. The most common injuries are simple sprains (31 to 53%), followed by fractures (24 to 27%), and contusions (12%). Blunt cardiac injury associated with snowboarding is extremely rare [4]. More than 90% of blunt cardiac trauma occurs in traffic accidents [1]. In other words, the snowboarding injuries in this patient are similar to injuries from high-energy trauma involving traffic accidents. Snowboarders on new equipment achieve high speeds when doing downhill snowboarding. It is possible that a high level of participation by novice snowboarders without protective gear could lead to severe extremity injury.

The second point is in regards to ECG abnormalities in blunt cardiac injury. The present case showed blunt trauma of his left atrium. The heart moves relatively freely in the anteroposterior direction. Hence, it often collides to the inside of sternal by external force such as a traffic accident. Therefore, typical blunt cardiac trauma occurs at the right ventricle. Blunt trauma of the left atrium is very rare [1]. It was reported that ECG abnormalities, increasing serum CK-MB and troponin concentrations, and echocardiographic findings were useful for diagnosing cardiac injury [5, 6]. In particular, ECG is an important screening test for patients with potential blunt cardiac injury. ECG abnormalities in blunt cardiac injury can include sinus tachycardia, other arrhythmias, new bundle branch block, or ST depression or elevation [7, 8]. In the present case, CRBBB was the only ECG abnormality detected. The right bundle branch is vulnerable to stretch by trauma [9]. The CRBBB pattern changed to a narrow QRS pattern after surgery in the present case, and this change may have been caused by blunt cardiac injury, even though the site of injury was the left atrium. Although CRBBB generally can be considered benign, in trauma, all ECG changes should be suspected to indicate cardiac injury until proven otherwise. To detect cardiac trauma in snowboarding accidents, an examination of an ECG is required in all patients who might have a bruised chest.

## Conclusions

The present case involved blunt trauma of the left atrium with CRBBB on ECG due to snowboarding trauma.
